# *CRLF2* and *IKZF1* abnormalities in Mexican children with acute lymphoblastic leukemia and recurrent gene fusions: exploring surrogate markers of signaling pathways

**DOI:** 10.1002/cjp2.211

**Published:** 2021-04-23

**Authors:** Dafné Moreno Lorenzana, María del Rocío Juárez Velázquez, Adriana Reyes León, Daniel Martínez Anaya, Adrián Hernández Monterde, Consuelo Salas Labadía, María del Pilar Navarrete Meneses, Marta Zapata Tarrés, Luis Juárez Villegas, Berenice Jarquín Ramírez, Rocío Cárdenas Cardós, Martha Herrera Almanza, Rogelio Paredes Aguilera, Patricia Pérez Vera

**Affiliations:** ^1^ Laboratorio de Genética y Cáncer Instituto Nacional de Pediatría Mexico City Mexico; ^2^ Cátedra CONACYT‐Instituto Nacional de Pediatría Mexico City Mexico; ^3^ Posgrado en Ciencias Biológicas Universidad Nacional Autónoma de México Mexico City Mexico; ^4^ Servicio de Oncología Instituto Nacional de Pediatría Mexico City Mexico; ^5^ Servicio de Hemato‐Oncología Hospital Infantil de México Federico Gómez Mexico City Mexico; ^6^ Especialidad en Citogenética Humana Instituto Nacional de Pediatría Mexico City Mexico; ^7^ Becaria de la Dirección General de Calidad y Educación en Salud Secretaría de Salud México Mexico City Mexico; ^8^ Servicio de Hematología Instituto Nacional de Pediatría Mexico City Mexico

**Keywords:** *CRLF2*, *IKZF1*, activated signaling pathways, acute lymphoblastic leukemia, primary rearrangements, Mexican children, *TCF3*‐*PBX1* concurrent with *P2RY8*‐*CRLF2*

## Abstract

The gene fusions *BCR*‐*ABL1*, *TCF3*‐*PBX1*, and *ETV6*‐*RUNX1* are recurrent in B‐cell acute lymphoblastic leukemia (B‐ALL) and are found with low frequency in coexistence with *CRLF2* (cytokine receptor‐like factor 2) rearrangements and overexpression. There is limited information regarding the *CRLF2* abnormalities and dominant‐negative *IKZF1* isoforms associated with surrogate markers of Jak2, ABL, and Ras signaling pathways. To assess this, we evaluated 24 Mexican children with B‐ALL positive for recurrent gene fusions at diagnosis. We found *CRLF2* rearrangements and/or overexpression, dominant‐negative *IKZF1* isoforms, and surrogate phosphorylated markers of signaling pathways coexisting with recurrent gene fusions. All the *BCR*‐*ABL1* patients expressed CRLF2 and were positive for pCrkl (ABL); most of them were also positive for pStat5 (Jak2/Stat5) and negative for pErk (Ras). *TCF3*‐*PBX1* patients with *CRLF2* abnormalities were positive for pStat5, most of them were also positive for pCrkl, and two patients were also positive for pErk. One patient with *ETV6*‐*RUNX1* and intracellular CRLF2 protein expressed pCrkl. In some cases, the activated signaling pathways were reverted *in vitro* by specific inhibitors. We further analyzed a *TCF3*‐*PBX1* patient at relapse, identifying a clone with the recurrent gene fusion, *P2RY8*‐*CRLF2*, rearrangement, and phosphorylation of the three surrogate markers that we studied. These results agree with the previous reports regarding resistance to treatment observed in patients with recurrent gene fusions and coexisting *CRLF2* gene abnormalities. A marker phosphorylation signature was identified in *BCR*‐*ABL1* and *TCF3*‐*PBX1* patients. To obtain useful information for the assessment of treatment in B‐ALL patients with recurrent gene fusions, we suggest that they should be evaluated at diagnosis for *CRLF2* gene abnormalities and dominant‐negative *IKZF1* isoforms, in addition to the analyses of activation and inhibition of signaling pathways.

## Introduction

B‐cell acute lymphoblastic leukemia (B‐ALL) is a complex disease with many gene abnormalities and disturbed functional processes; thus, a plethora of genetic lesions and heterogeneous abnormal signaling pathways can be detected [1, 2]. The fusions *BCR*‐*ABL1*, *TCF3*‐*PBX1*, *ETV6*‐*RUNX1*, and *KMT2A*‐variant are recurrent genetic abnormalities with biological significance in B‐ALL [3]. These gene fusions are initial leukemogenic events that confer self‐renewal properties to hematopoietic stem cells or lymphoid progenitors [1].

The novel *BCR*‐*ABL1*‐like B‐ALL subtype is recognized as a high‐risk subgroup characterized by alterations in kinases or their receptors, resulting in the constitutive activation of signaling pathways. Recurrent gene fusions and *BCR*‐*ABL1*‐like are mutually exclusive [4].

*BCR*‐*ABL1*‐like patients present *CRLF2* (cytokine receptor‐like factor 2) gene rearrangements in 60% of the cases. The most frequent rearrangements are the *P2RY8*‐*CRLF2* fusion, generated by deletion of the pseudoautosomal region PAR1 and present in 25.8% of the *BCR*‐*ABL1*‐like patients, and the *IGH*‐*CRLF2* rearrangement, produced by t(X;14)(p22;q32) or t(Y;14)(p11;q32) and observed in 3.5% of *BCR*‐*ABL1*‐like patients [5]. Both abnormalities result in the binding of transcriptional control elements to the coding sequence of *CRLF2*, leading to overexpression of the encoded protein [2, 6, 7]. Another hallmark of the *BCR*‐*ABL1*‐like subgroup is the high frequency of abnormalities in the *IKZF1* gene (IKZF1a), which are mainly deletions or dominant‐negative isoforms [4].

The coexistence of *CRLF2* rearrangements and overexpression with recurrent B‐ALL abnormalities, such as *ETV6*‐*RUNX1*, *BCR*‐*ABL1*, iAMP21, or hyperdiploidy, has been reported. CRLF2 overexpression occurs more frequently in children with hyperdiploidy (19%), and *P2RY8*‐*CRLF2* fusion is present in patients with iAMP21 (20%) [8, 9, 10, 11]. Although less frequent, the coexistence of *CRLF2* rearrangements with the *BCR*‐*ABL1* fusion has been observed in adults [10, 12, 13, 14]. In children, *CRLF2* rearrangements have been described coexisting with *ETV6*‐*RUNX1*, *KMT2A*‐*AFF1*, and only in one patient with *TCF3*‐*PBX1* [14, 15, 16, 17]. Notably, *CRLF2* rearrangements concurrent with *BCR*‐*ABL1* confer to patients' resistance to the ABL pathway inhibitors; furthermore, some of these patients have been diagnosed as *BCR*‐*ABL1*‐like [18]. Interestingly, the coexistence of recurrent gene fusions with *CRLF2* rearrangements in the same blast has been observed mostly in patients of Hispanic and Mexican American origin [18, 19].

The *IKZF1* gene encodes the transcription factor Ikaros; importantly, in leukemic cells, one of its targets is the *CRLF2* promoter [20]. The most frequent somatic abnormality in B‐ALL is *IKZF1* deletion, which is present in 83% of *BCR*‐*ABL1* patients [21]. *IKZF1* deletions and point mutations are also frequent among *BCR*‐*ABL1*‐like patients and present in 68% of cases [22]. In contrast, IKZF1a is less common in cases with gene fusions such as *TCF3*‐*PBX1* (1–4%) [23, 24] and *ETV6*‐*RUNX1* (3–6%) [24, 25, 26].

As mentioned above, *CRLF2* rearrangements, concurrent with other recurrent gene fusions, have been rarely described. It is possible to identify recurrent genetic fusions in combination, either in the same cell or in separate cells of the same patient [14, 18, 27]. These observations prompted us to describe a group of Mexican children with B‐ALL and recurrent gene fusions, searching for *CRLF2* rearrangements and overexpression, dominant‐negative *IKZF1* isoforms (Ik6, Ik8, and IKZF1a), and surrogate markers for Jak2, ABL, and Ras signaling pathways. All these features were selected as they are characteristic of *BCR*‐*ABL1*‐like patients [22].

## Materials and methods

### Patients

One hundred and thirty‐eight bone marrow samples from B‐ALL children at diagnosis were collected at the Instituto Nacional de Pediatría and Hospital Infantil de México Federico Gómez in Mexico City, Mexico. Diagnosis of B‐ALL was based on morphological characteristics of bone marrow smears together with the immunophenotype analysis of leukemic cells by flow cytometry. Mononuclear cells from bone marrow samples were isolated and used for obtaining suitable biologic material for the genetic and flow cytometry analyses. The detection of gene fusions was performed by reverse transcription polymerase chain reaction (RT‐PCR) using a diagnostic kit for 28 leukemia gene fusions (HemaVision RT‐PCR, Risskov, Denmark). Genetic and flow cytometry studies were performed in patients with fusions when RNA and/or cells were available. Informed consent was obtained from the guardians of patients according to the Declaration of Helsinki. The project was approved and it followed the guidelines of the Research and Institutional Ethics Committees of the participant Institutions.

### Determination of *CRLF2* transcript expression

RNA was extracted from bone marrow mononuclear cells using the RNeasy kit (Qiagen, Hilden, Germany) according to the manufacturer's instructions. cDNA was obtained by standard methods (Invitrogen, Waltham, MA, USA). Relative quantification of *CRLF2* transcripts was determined by real‐time RT‐PCR, using a LightCycler 2.0 Instrument (Roche Applied Science, Mannheim, Germany) with TaqMan gene expression probes from the Universal Probe Library System (Roche Applied Science). The primer sets that we used for *CRLF2* (NM_022148) were forward primer 5′‐AGCGACTGGTCAGAGGTGA‐3′ and reverse primer 5′‐AATTTGGACAGCTTTGGTTTG‐3′; the probe we used was #53. Quantification of transcripts was calculated by the ΔΔCt method using the *GUSβ* gene as an endogenous control for data normalization. The primer sets for *GUSβ* (NM_000181.3) were forward primer 5′‐CGCCCTGCCTATCTGTATTC‐3′ and reverse primer 5′‐TCCCCACAGGGAGTGTGTGTAG‐3′; the probe was #57. Gene expression was analyzed in duplicate. The *CRLF2* high expression cut‐off value was determined in an independent cohort of 70 ALL patients without recurrent primary alterations. The normalized numerical values of relative expression were divided into high or low *CRLF2* expression groups (quartile 1 − quartile 2 versus quartile 3 − quartile 4) and the cut‐off value (14.3) was determined by GraphPad Prism 8 software (GraphPad, San Diego, CA, USA) [20, 28]. Statistical analysis with analysis of variance (ANOVA) showed that *CRLF2* expression in the two groups was significantly different (*p* = 0.05). It must be noted that in the mentioned cohort the first patient above the cut‐off value was positive for *IGH*‐*CRLF2*, and no patient with this rearrangement was found below this value.

### Analysis of the *P2RY8‐CRLF2* deletion

The deletion was detected by RT‐PCR based on the methods described by Palmi *et al* [29]. To detect the deletion in samples with minor cell subclones, the PCR product of the first reaction was reamplified using the same set of primers.

### Analysis of Ik6 and Ik8 transcripts

The transcripts of the dominant‐negative isoforms Ik6 and Ik8 were detected by nested RT‐PCR, based on the methods described by Iacobucci *et al* [30, 31]. The expected sizes of the PCR products of the Ik8 and Ik6 isoforms are 390 and 255 base pairs, respectively.

### Determination of CRLF2 protein expression by flow cytometry

In patients from whom cells were available, flow cytometry studies were performed. CRLF2 protein expression was assessed using an allophycocyanin (APC)‐conjugated monoclonal antibody that recognizes the extracellular domain (Becton Dickinson, Franklin Lakes, NJ, USA) [32]. Bone marrow cells were stained with cell surface monoclonal antibodies: CD45 Amcyan, CD34 PECy7, CD19 PerCP, and CD10 PE (Becton Dickinson). To identify cell surface CRLF2 or intracellular CRLF2, two different staining conditions were used: CRLF2 antibody incubation in fresh cells and CRLF2 antibody incubation after fixation, and permeabilization with BD Cytofix/Cytopermä (Becton Dickinson). The antibody concentration and conditions for the assay were performed as per the manufacturer's recommendations. Flow cytometry was performed using a BD FACSVerse Cell Analyzer System and data were analyzed by FlowJo vX software (Becton, Dickinson and Company, Ashland, OR, USA).

### Identification of kinase alterations and inhibition assay by phosphoflow cytometry

To identify Jak2/Stat5, ABL, and Ras pathway alterations in ALL patient samples, fresh cells from bone marrow were stimulated with 200 ng/ml thymic stromal lymphopoietin (TSLP) (PeproTech, Cranbury, NJ, USA) for 30 min at 37 °C, and stained with the following cell surface monoclonal antibodies: CD45 Amcyan, CD34 PECy7, CD19 PerCP, and CD10 PE (Becton Dickinson, USA) [32]. Then, the cells were fixed with BD Phosflow Fix Buffer I, permeabilized with BD Phosflow Perm Buffer III (Becton Dickinson), and incubated with monoclonal antibodies to identify phosphorylated targets of each pathway: Stat5 (pY694) Pacific Blue to identify Jak2, CrkL (pY207) AF488 for ABL, and Erk 1/2 (pT202/pY204) AF488 for Ras. In cases with an adequate number of cells, the abnormal pathway activity was selectively inhibited *in vitro* for 30 min with ruxolitinib (5 nm) (Selleckchem, Houston, TX, USA) to inhibit Jak2/Stat5 pathway, and with imatinib and/or dasatinib (5 μm) (Selleckchem) to prevent ABL abnormal activation. The antibody concentration and conditions for each assay were performed based on the manufacturer's recommendations. Flow cytometry was performed using a BD FACSVerse Cell Analyzer System and data were analyzed by FlowJo vX software.

### FISH studies in a patient with *TCF3‐PBX1* at relapse

In this patient, a previous conventional cytogenetic analysis with GTG bands was performed to determine the t(1;19)(q25;p13). The LSI 1ptel/1p36/1q35 and ToTelVysion mix #14 including p and q subtelomeres of chromosome 19 by Vysis‐Abbott (Abbott Park, IL, USA) probes were used to detect the t(1;19) by fluorescence *in situ* hybridization (FISH). The *P2RY8*‐*CRLF2* fusion was detected by single‐sequence double‐color probes (Cytocell, Cambridge, UK).

## Results

### *CRLF2* rearrangements

One hundred and thirty‐eight bone marrow samples were collected from B‐ALL patients at diagnosis; 24 patients presented recurrent gene fusions (Table 1). We analyzed *CRLF2* expression, the *P2RY8*‐*CRLF2* fusion, and cell surface and intracellular CRLF2 protein in these patients. Of these 24 patients, 5 were positive for the *P2RY8*‐*CRLF2* fusion, 8 presented *CRLF2* overexpression, CRLF2 protein was detected on the cell surface in 11, and 14 cases had intracellular CRLF2 protein; 3 of these patients were not analyzed for cell surface protein. A total of 19 of 24 patients had the *P2RY8*‐*CRLF2* fusion, *CRLF2* overexpression, or CRLF2 protein expression, and the following recurrent gene fusions: 6 of 6 *BCR*‐*ABL1*‐positive patients, 9 of 12 cases with *TCF3*‐*PBX1*, and 4 of 6 patients with *ETV6*‐*RUNX1* (Table 1).

**Table 1:** CRFL2 abnormalities in children with B‐ALL and recurrent gene fusions.

Patients	Primary abnormality	*P2RY8‐CRLF2*	*CRLF2* gene expression	CRLF2 protein (%)		Ik6 and Ik8 Isoforms
				On cell surface	Intracellular	
L1	*BCR*‐*ABL1*	Neg	High	1.54	26	Ik6, Ik8
L2		Neg	High	4.18	45.9	Neg
L3		Neg	High	ND	ND	Neg
L4		Neg	High	ND	ND	Ik6
L5		Neg	Low	13.6	69.5	Ik6, Ik8
L6		Neg	Low	ND	100	Ik6
L7	*TCF3*‐*PBX1*	Pos	High	1.7	64.8	Neg
L8		Neg	High	2	90.3	Ik6, Ik8
L9		Neg	Low	3	34	Ik8
L10		Pos	Low	23.2	90.8	Ik8
L11		Pos	ND	1.7	91.5	Neg
L12		Neg	Low	10.7	46.7	Neg
L13		Neg	Low	3.5	63.2	Ik6, Ik8
L14		Pos	Low	ND	92	Neg
L15		Neg	High	ND	Neg	Ik6
L16		Neg	Low	Neg	Neg	Ik6
L17		Neg	Low	ND	ND	Neg
L18		Neg	Low	ND	ND	Neg
L19	*ETV6*‐*RUNX1*	Pos	Low	ND	ND	Ik6, Ik8
L20		Neg	High	ND	ND	Neg
L21		Neg	Low	91.7	98.8	Neg
L22		Neg	Low	ND	100	Neg
L23		Neg	Low	ND	ND	Ik6, Ik8
L24		Neg	Low	ND	ND	Ik6, Ik8

Cells in gray represent positive results.

ND, not determined; Neg, negative; Pos, positive.

### *P2RY8*‐*CRLF2* fusion

None of the patients with *BCR*‐*ABL1* presented the *P2RY8*‐*CRLF2* fusion; however, 4 of 12 patients with *TCF3*‐*PBX1* and 1 of 6 cases with *ETV6*‐*RUNX1* were positive.

### *CRLF2* expression

**Figure 1:**
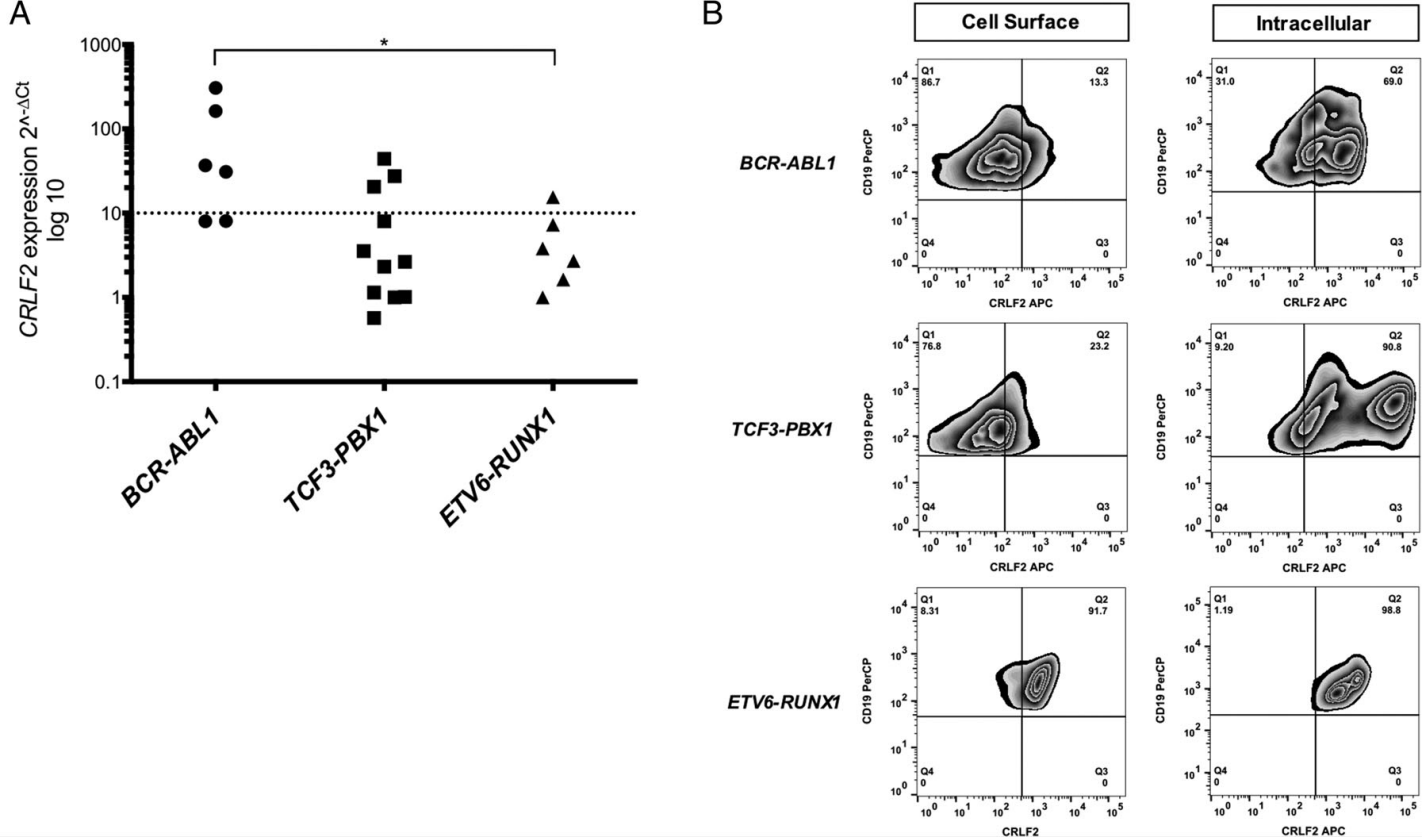
(A) *CRLF2* expression in patients with B‐ALL and gene fusions. The dispersion of the transcript levels is shown; above the line was considered high expression. (B) Flow cytometry analysis of intracellular and extracellular CRLF2 protein in the blast population of patients with gene fusions. * p<0.05.

*CRLF2* overexpression was found in 4 of 6 patients with *BCR*‐*ABL1*, 3 of 12 with *TCF3*‐*PBX1*, and 1 of 6 with *ETV6*‐*RUNX1* (Table 1 and Figure 1A). Thus, *CRLF2* overexpression was more frequent in patients with *BCR*‐*ABL1* than in those with other fusions (*p = 0.0178, one‐way ANOVA test, GraphPad Prism 8 software).

### CRLF2 protein

The CRLF2 protein was determined in patients with available samples (16/24, 66%) (Table 1). Interestingly, most patients presented CRLF2 protein in blasts, on the cell surface, and within cells (Table 1 and Figure 1B). In *BCR*‐*ABL1* patients, cell surface CRLF2 protein was present in a lower percentage of blasts (1.54–13.6%) than the intracellular protein (26–100%). Similar results were observed in the *TCF3*‐*PBX1* patients; cell surface CRLF2 protein was present in 1.7–23.2% of blasts and the intracellular protein was present in a high proportion (34–92%). Within the *ETV6*‐*RUNX1* group, patient L21 presented a high percentage of blasts positive for both CRLF2 cell surface and intracellular protein (91.7 and 98.8%) and patient L22 showed 100% of blasts with intracellular CRLF2 protein.

All the patients positive for cell surface and intracellular CRLF2 protein were analyzed for *CRLF2* expression and presented high or low expression levels (Table 1).

### Surrogate markers of signaling pathways

Fifteen patients were analyzed for surrogate markers of Jak2, ABL, and Ras signaling pathways (Table 2). All *BCR*‐*ABL1* patients analyzed for these surrogate markers were positive for CRLF2 protein. Most of the *BCR*‐*ABL1* patients (3/4) were positive for Stat5 phosphorylation. As expected, all these patients were positive for phosphorylation of the main target of the ABL pathway, CrkL, and were negative for Erk1/2, the Ras pathway's main target.

**Table 2:** Analysis of surrogate markers of Jak2/Stat5, ABL, and Ras pathways in patients with B‐ALL and recurrent gene fusions.

Patients	Primary abnormality	Pathway activation		
		Stat5 (pY694)	CrkL (pY207)	Erk 1/2 (pT202/pY204)
L1	*BCR*‐*ABL1*	Low	Low	Neg
L2		Low	Low	Neg
L5		Med	Med	Neg
L6		Neg	Low	Neg
L7	*TCF3*‐*PBX1*	Low	Low	Neg
L8		High	Low	Neg
L9		Med	Low	Neg
L10		High	Med	Med
L11		Med	Low	Low
L12		Low	Low	Neg
L13		Med	Low	Neg
L14*		Low	Neg	ND
L15		Neg	Neg	Neg
L21^†^	*ETV6*‐*RUNX1*	ND	Low	ND
L22		Neg	Neg	Neg

Cells in gray represent positive results.

Low, 1‐30%; Med, 31‐60%; High, 61‐100%; ND, not determined; Neg, negative.

^*^ Inhibited with ruxolitinib.

^†^ Inhibited with imatinib and dasatinib.

**Figure 2:**
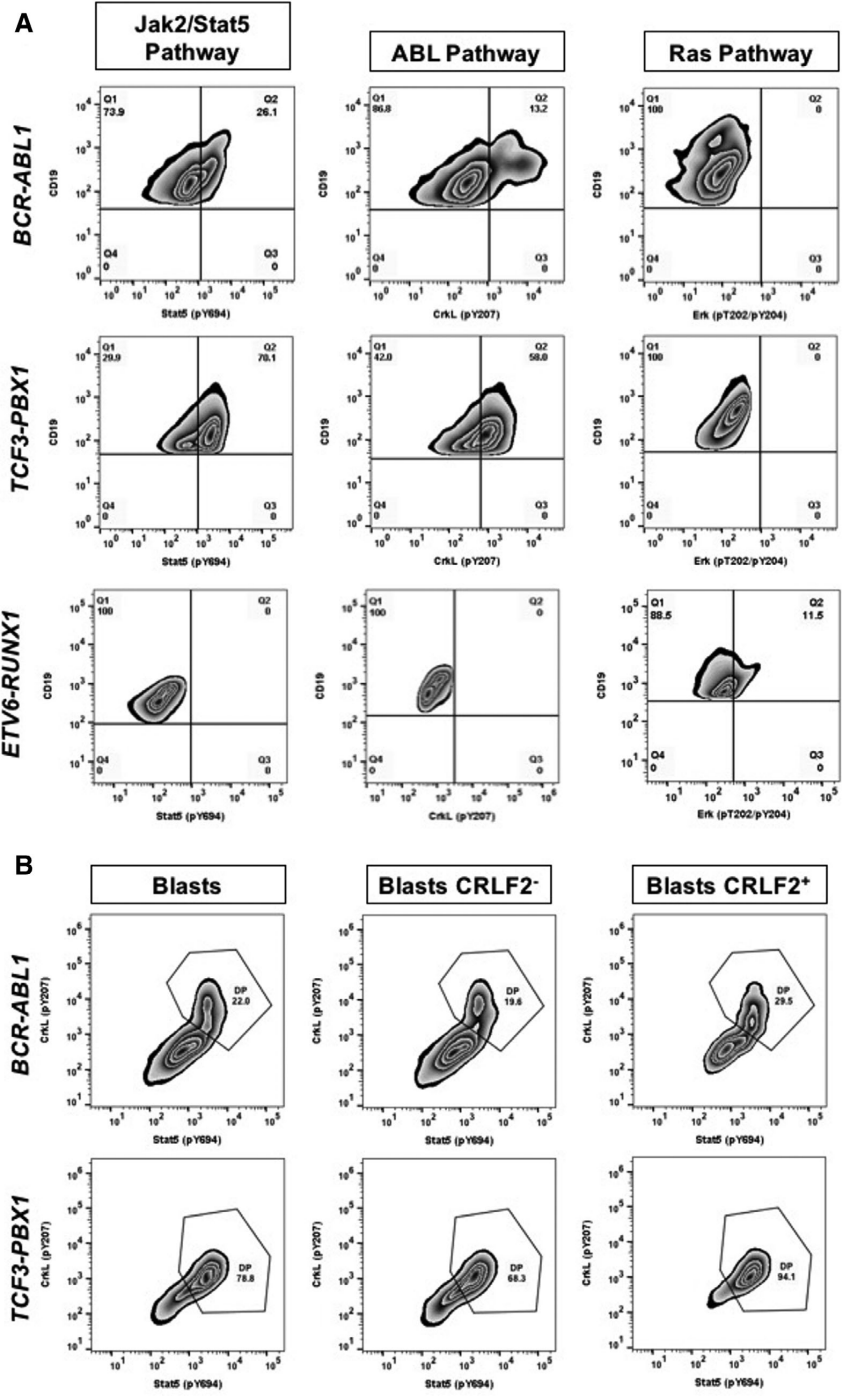
(A) Surrogate markers of the Jak2/Stat5, ABL, and Ras signaling pathways analyzed by phosphoflow assay in the blast populations of patients with coexistence of *CRLF2* rearrangements and B‐ALL gene fusions. (B) Coexistence of phosphorylated markers of the Jak2/Stat5 and ABL (double positive, DP) signaling pathways through the phosphoflow assay in the blast populations of *BCR*‐*ABL1*‐ and *TCF3*‐*PBX1*‐positive patients.

Nine patients in the *TCF3*‐*PBX1* group were analyzed; eight were positive for CRLF2 protein and for Stat5 phosphorylation. Unexpectedly, seven of nine cases were positive for CrkL and two were positive for Erk 1/2 (Table 2 and Figure 2A). Interestingly, one *TCF3*‐*PBX1* patient (L15) negative for CRLF2 protein was negative for the analyzed surrogate markers. In one patient with *TCF3*‐*PBX1* (L14), the Stat5 phosphorylation was reverted *in vitro* using the specific inhibitor ruxolitinib (Table 2 and supplementary material, Figure S1).

Two patients with *ETV6*‐*RUNX1*, who expressed CRLF2 protein, were analyzed for surrogate markers of the ABL pathway; patient L21 was positive for CrkL phosphorylation, reverted *in vitro* with imatinib and dasatinib (Table 2 and supplementary material, Figure S1). None of the patients of this subtype were positive for Stat5 phosphorylation.

### *IKZF1* dominant‐negative isoform transcripts

We found a high frequency of dominant‐negative *IKZF1* isoform transcripts in all the patients (13/24, 54.1%). The Ik6 and Ik8 isoforms were observed particularly in four of six *BCR*‐*ABL1* cases, either in the same patient or independently. The isoforms were present in 6 of 12 cases with *TCF3*‐*PBX1* and 3 of 6 cases with *ETV6*‐*RUNX1* (Table 1).

### Patients' evolution

Patients' follow‐up time is less than 3 years. At present, three of the patients with *CRLF2* rearrangements or overexpression and phosphorylated surrogate markers have developed early relapses and died; one of them was *BCR*‐*ABL1* positive (L2) and two showed *TCF3*‐*PBX1* fusion (L7 and L13). It was possible to study a 2‐year‐old male diagnosed with B‐ALL at relapse, who was positive for *TCF3*‐*PBX1*. The sample at diagnosis was not available to evaluate *CRLF2* rearrangements, overexpression, or signaling pathways. The patient was treated based on the St. Jude Total Therapy XIII‐B protocol and relapsed 2.4 years later presenting the same fusion as at diagnosis. We determined by FISH in metaphases the coexistence of *TCF3*‐*PBX1* and *P2RY8*‐*CRLF2* fusions in the same cell at relapse (see supplementary material, Figure S2). The patient was positive for CRLF2 surface protein (15%) and phosphorylation of Stat5 (93%), CrkL (90%), and Erk 1/2 (66%). Ruxolitinib and dasatinib inhibited two signaling pathways; Ras signaling inhibition was not determined (see supplementary material, Figure S2).

## Discussion

We identified 24 B‐ALL Mexican children positive for recurrent gene fusions: *BCR*‐*ABL1*, *TCF3*‐*PBX1*, or *ETV6*‐*RUNX1*. These patients were assessed for *CRLF2* rearrangements, transcript and protein, dominant‐negative *IKZF1* isoforms, and phosphorylation of surrogate markers of Jak2, ABL, and Ras signaling pathways.

### Concurrence of *CRLF2* abnormalities with B‐ALL gene fusions

The co‐occurrence of B‐ALL recurrent gene fusions with *CRLF2* rearrangements and overexpression is an uncommon finding and only 20 patients have been previously reported (see supplementary material, Table S1). Most of these cases have been detected by screening *CRLF2* overexpression and/or rearrangements in B‐ALL children or adults. *BCR*‐*ABL1* is the most frequent fusion found concurrent with *CRLF2* abnormalities; this condition confers an aggressive disease progression with clones resistant to treatment with ABL inhibitors [18, 19].

*CRLF2* rearrangements and overexpression are commonly determined in the *BCR*‐*ABL1*‐like B‐ALL subtype, but not in B‐ALL patients with recurrent gene fusions, who are not screened for *CRLF2* abnormalities [10, 12, 13, 14, 15, 16, 17, 18, 19]. This exclusion criterion could underestimate the frequency of patients with the coexistence of recurrent gene fusions, and *CRLF2* rearrangements and overexpression.

The incidence of B‐ALL among populations, the frequencies of genetic variants for B‐ALL susceptibility, and recurrent gene fusion rates are influenced by ethnic differences [33, 34, 35]. According to our literature review, the coexistence of *BCR*‐*ABL1* fusion with *CRLF2* rearrangements and overexpression could be more common in Mexican American patients (see supplementary material, Table S1). Therefore, it is essential to consider that ethnicity could influence these findings [13, 18, 19]. Another related fact is that the incidence of childhood B‐ALL in Mexicans (79.8/million) is much higher compared to other populations [36, 37], and Hispanic children have worse outcomes and the lowest survival rates [33, 38, 39]. In addition, overrepresentation of *CRLF2* rearrangements and overexpression has been observed in the B‐ALL Hispanic population [7]. Besides the natural history of each genetic subtype of B‐ALL, the Mexican population's genetic background may impact patients' recurrence with the coexistence of *CRLF2* abnormalities and recurrent gene fusions [7, 18, 19]. Further studies in a higher number of Mexican B‐ALL patients and in non‐Hispanic B‐ALL populations must be performed to prove this hypothesis.

### Surrogate markers to evaluate signaling pathway activation in patients with *CRLF2* abnormalities and recurrent gene fusions

It has been observed that *in vitro* TSLP stimulation of blasts carrying *CRLF2* rearrangements or overexpression activates multiple signaling molecules, including Stat5, Erk, and Src [40]. In the context of B‐ALL recurrent gene fusions, coexisting with *CRLF2* abnormalities, we found phosphorylation of Stat5, CrkL and, Erk.

Three *BCR*‐*ABL1*‐positive patients were positive for CrkL and Stat5 phosphorylation in the same group of blasts (Figure 2B). These findings could be associated with *CRLF2* overexpression through the activation of CRLF2‐IL7Ra by the TSLP ligand, which induces the Jak2/Stat5 pathway increasing blast proliferation and survival. Another plausible option is that BCR‐ABL1 p190 protein, the most frequent isoform in ALL patients, contributes to the direct activation of CrkL, but also of Stat5 through Jak2 [41]. One *BCR*‐*ABL1* patient (L6) was negative for CrkL activation, and a possible explanation is that both *CRLF2* overexpression and *JAK2* mutation are required for phosphorylation of Stat5; unfortunately, the mutational status of *JAK2* was not determined [40]. Our results suggest crosstalk and a synergistic effect between BCR‐ABL1 and CRLF2 to activate the Jak2/Stat5 pathway [42, 43], which could be prone to induce chemoresistance [18].

One *ETV6*‐*RUNX1* patient was positive for *P2RY8*‐*CRLF2* fusion, but not for *CRLF2* overexpression (L19). This fusion might be present in a minor clone, not being enough to allow the detection of *CRLF2* overexpression; unfortunately, a sample for FISH analysis was not available to determine the size of clones [44]. As in *BCR*‐*ABL1*‐positive patient L6, patient L22 presented intracellular CRLF2 protein in a high percentage of the blasts; however, it was negative for Stat5 phosphorylation. This is probably related to the absence of a *JAK2* mutation [40]. Patient L21 was positive for CrkL phosphorylation. This finding is not common in the *ETV6*‐*RUNX1* context. Nevertheless, we cannot discard a genetic abnormality in a member of the ABL pathway. An *ETV6*‐*RUNX1*‐positive patient with a *BCR*‐*ABL1* fusion as a secondary abnormality has already been reported [45]. Although this fusion is not present in the L21 patient, other ABL‐type mutations may cause the pathway activation.

Among the eight *TCF3*‐*PBX1* patients with CRLF2 protein, four presented *P2RY8*‐*CRLF2* fusion and Stat5 phosphorylation; this finding suggests that Jak2‐Stat5 activation occurs through the CRLF2‐IL7Ra‐TSLP pathway. Besides, seven of eight patients with *CRLF2* expression showed CrkL phosphorylation. In a conditional *E2a*‐*Pbx1* mice model, it has been reported that this fusion is responsible for Src protein‐tyrosine kinase family overexpression. These kinases are downstream targets of functional pre‐BCR (B‐cell receptor), a distinctive feature of human *TCF3*‐*PBX1* leukemia, and two of its main targets after pre‐BCR stimulation are CrkL and PLCg2 [46, 47]. Therefore, the two patients negative for CrkL phosphorylation might be pre‐BCR negative [48, 49]. Besides the pathway activation findings, these patients could present other mutations as part of their leukemia's natural history. Mutations in several members of the Jak/Stat pathway (*Jak1*, *Jak3*, *Ptpn11*, and *Il7r* genes) and the Ras pathway (*Kras* and *Nras*) have been reported in the *E2a*‐*Pbx1* mouse model [1]. Interestingly, patients L10 and L11 showed phosphorylation for all the analyzed markers, including Erk; this characteristic is related to aggressiveness as, in the mouse model, *Ras* mutations conferred a shorter latency to develop leukemia [1].

It is impossible to ascertain if these results will be statistically significant, given the limited number and short time of patients' follow‐up; however, our findings could be relevant for treatment selection. To investigate this possibility, we studied a relapse patient positive for *TCF3*‐*PBX1*. Our results are in line with other reports regarding resistance to treatment in patients with B‐ALL and the coexistence of *BCR*‐*ABL1* and *CRLF2* abnormalities [18]. A similar situation could be present in this relapsed patient and the other *TCF3*‐*PBX1*‐positive cases we examined (Tables 1 and 2).

In T‐cell acute lymphoblastic leukemia (T‐ALL) patients, Stat5 phosphorylation was induced by TSLP, although CRLF2 protein was localized intracellularly [32]. It is unknown if there is activation through the intracellular protein, but it is possible that the surface receptor, even in low quantity, could be capable of inducing the signaling pathway through a low amount of ligand entering into the cell [32, 50]. Considering that CRLF2 surface protein was present in a low fraction of blasts, proteomic characterization of intracellular CRLF2 is required to determine if a mutation prevents the protein from reaching the cell surface or if it is a soluble isoform [51, 52, 53]. The transcript was detected at high or low expression levels in almost all the patients with the cell surface or intracellular CRLF2 protein. The cases with low expression could be explained by the presence of mutations or single‐nucleotide variants modifying the *CRLF2* transcript translation rate or half‐life [54, 55].

A group of patients showed high expression of *CRLF2* but were negative for *P2RY8*‐*CRLF2* fusion. The *CRLF2* overexpression may have been caused by a nondetermined *IGH*‐*CRLF2* rearrangement, *CRLF2* or *JAK2* mutations, or Ck2 kinase overactivity [46]. Also, it has been reported that expression of the dominant‐negative IKZF1 isoforms, Ik6 and Ik8, can cause overexpression of *CRLF2* as *IKZF1* acts as a negative transcriptional regulator of this gene. The expression of *CRLF2* is negatively regulated through epigenetic changes produced by IKZF1; thus, the loss of function of IKZF1 is in part responsible for the high expression of *CRLF2*. In our patients, the dominant‐negative IKZF1 isoforms were particularly frequent (54%) [20, 47].

**Figure 3:**
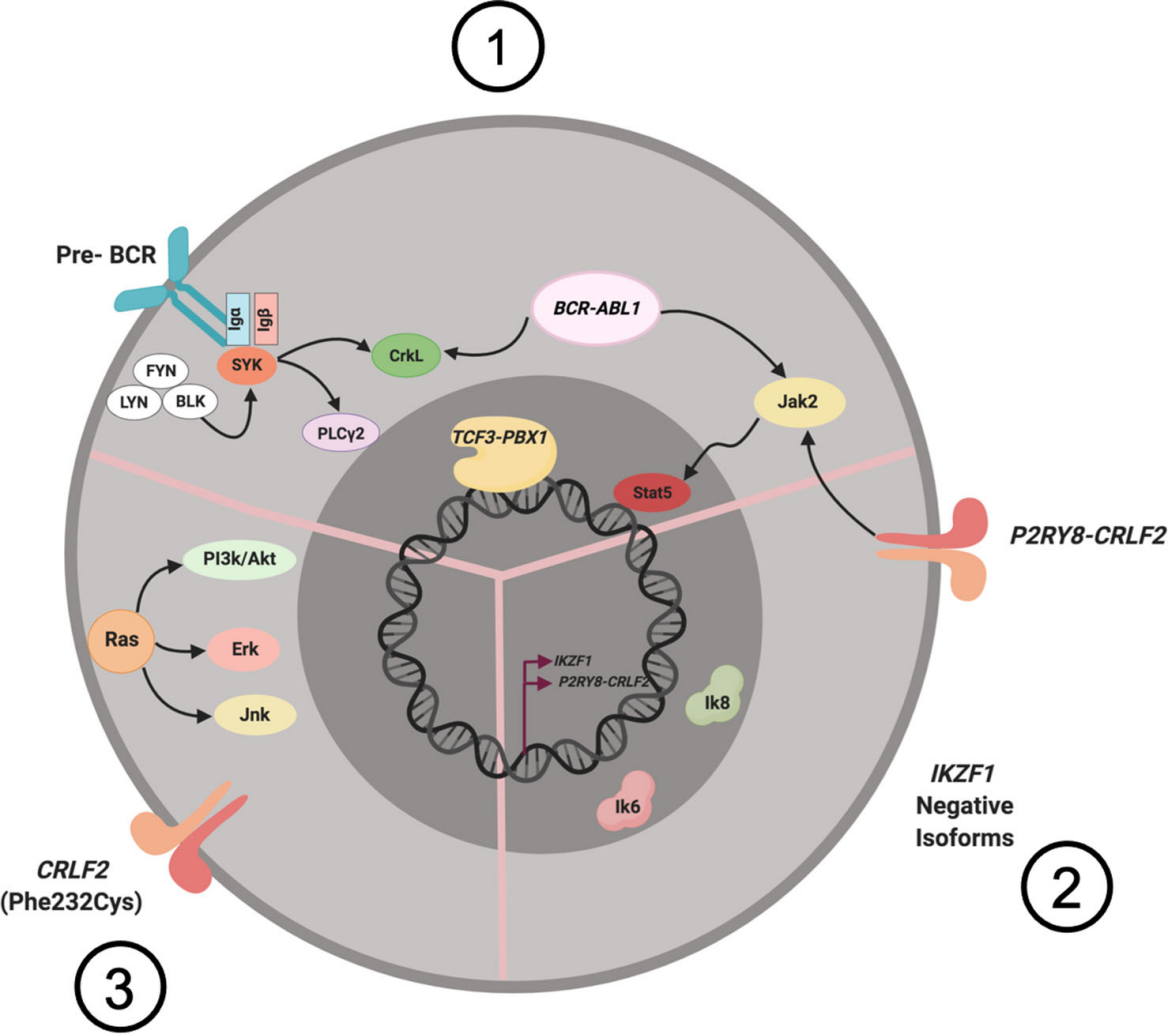
Three‐step model of B‐ALL pathogenesis. This model postulates (1) an initiating genetic lesion, in this case *TCF3*‐*PBX1* or *BCR*‐*ABL1*, that confers self‐renewal properties to hematopoietic stem cell or lymphoid progenitors; (2) a second lesion, such as *IKZF1* deletion or *P2RY8*‐*CRLF2* fusion, causing differentiation block at progenitor B‐cell level; and (3) requirement for a third class cooperating mutation to fully transform leukemia cells, affecting pathways such as cytokine receptors (*CRLF2* mutation) and/or Ras signaling activation.

Here, we report a new group of B‐ALL Mexican children with recurrent gene fusions in coexistence with *CRLF2* rearrangements and/or overexpression, and with phosphorylation of surrogate markers of Jak2, ABL, or Ras signaling pathways. In contrast to previously reported cases, most of our patients are *TCF3*‐*PBX1* positive. In *BCR*‐*ABL1* and *TCF3*‐*PBX1* patients, a signature of signaling pathway marker phosphorylation was identified, where gene fusions are the initial genetic alteration and confer self‐renewal properties to lymphoid progenitors [1, 48]. We observed second lesions affecting B‐cell development's essential transcription factors, such as the dominant‐negative *IKZF1* isoforms or *P2RY8*‐*CRLF2* fusion. Finally, *CRLF2* gene alterations or Ras pathway activation were detected as a third class of cooperating lesions to fully transform leukemia cells and affecting functions such as cytokine receptors and associated kinases [1, 27, 56]. Thus, we propose for our patients this multistep model of B‐ALL pathogenesis following the findings previously reported in cell lines and mice models [1, 40] (Figure 3).

As stated in the *TCF3*‐*PBX1*‐positive patient at relapse, the analyses of *CRLF2* rearrangements and expression, in addition to phosphorylation of surrogate markers of diverse signaling pathways, contribute relevant information to identify cell clones that could be resistant to treatment and are prone to be treated with specific inhibitors of Src, Ras, Ck2, and/or Jak2. These inhibitors could be combined with conventional chemotherapy to reduce leukemic cells' viability [57] or could be used to sensitize the cells to commonly used therapeutic agents [58, 59]. To obtain useful information for B‐ALL patients with recurrent gene fusions, we suggest assessing them at diagnosis for *CRLF2* gene abnormalities, activation of signaling pathways, and *in vitro* inhibition.

## Author contributions statement

DML conceived the study; designed, performed, and interpreted the flow cytometry experiments; drafted the manuscript; and designed the figures. MdRJV conceived the study; designed, performed, and interpreted *CRLF2* molecular experiments; drafted the manuscript; and designed the figures. ARL designed, performed, and interpreted molecular experiments (*IKZF1*). DMA designed and performed molecular (*CRLF2* and *IKZF1*) and FISH experiments; partial results of this study are part of his Master's degree dissertation (Posgrado en Ciencias Biológicas, Universidad Nacional Autónoma de México). AHM performed *CRLF2* molecular experiments. CSL analyzed the data and worked on the manuscript. MdPNM supported results interpretation and worked on the manuscript. MZT and LJV diagnosed the patients and obtained clinical data. BJR performed FISH and cytogenetic analysis; partial results of this study are part of her Specialist in Human Cytogenetics Thesis (Instituto Nacional de Pediatría, Secretaría de Salud). RCC and RPA recruited patients. MHA acquired clinical data and developed a database. PPV conceived the study, interpreted results, wrote the manuscript, and designed the tables.

## Supporting information


**Figure S1.** Phosphoflow analysis of the *in vitro* inhibition assay of Jak2/Stat5 and ABL pathways in blast populations from *TCF3*‐*PBX1* and *ETV6*‐*RUNX1* patients
**Figure S2.** Patient with *TCF3*‐*PBX1* at relapse
**Table S1.** B‐ALL patients with coexistence of *CRLF2* abnormalities and gene fusions reported in the literature and in this study
