# Supplementary material for: CRLF2 and IKZF1 abnormalities in Mexican children with acute lymphoblastic leukemia and recurrent gene fusions: exploring surrogate markers of signaling pathways
Source: J Pathol Clin Res. 2021 Apr 23;7(4):410–21. doi: 10.1002/cjp2.211 (PMC8185361; doi:10.1002/cjp2.211)

***CRLF2* and *IKZF1* abnormalities in Mexican children with acute lymphoblastic leukemia and recurrent gene fusions: exploring surrogate markers of signaling pathways**

D Moreno Lorenzana *et al*. *J Pathol Clin Res* DOI: 10.1002/cjp2.211

**Supplementary Material**

**Figure S1.** Phosphoflow analysis of the *in vitro* inhibition assay of Jak2/Stat5 and ABL pathways in blast populations from *TCF3-PBX1* and *ETV6-RUNX1* patients

**Figure S2.** Patient with *TCF3-PBX1* at relapse

**Table S1.** B-ALL patients with coexistence of *CRLF2* abnormalities and gene fusions reported in the literature and in this study.


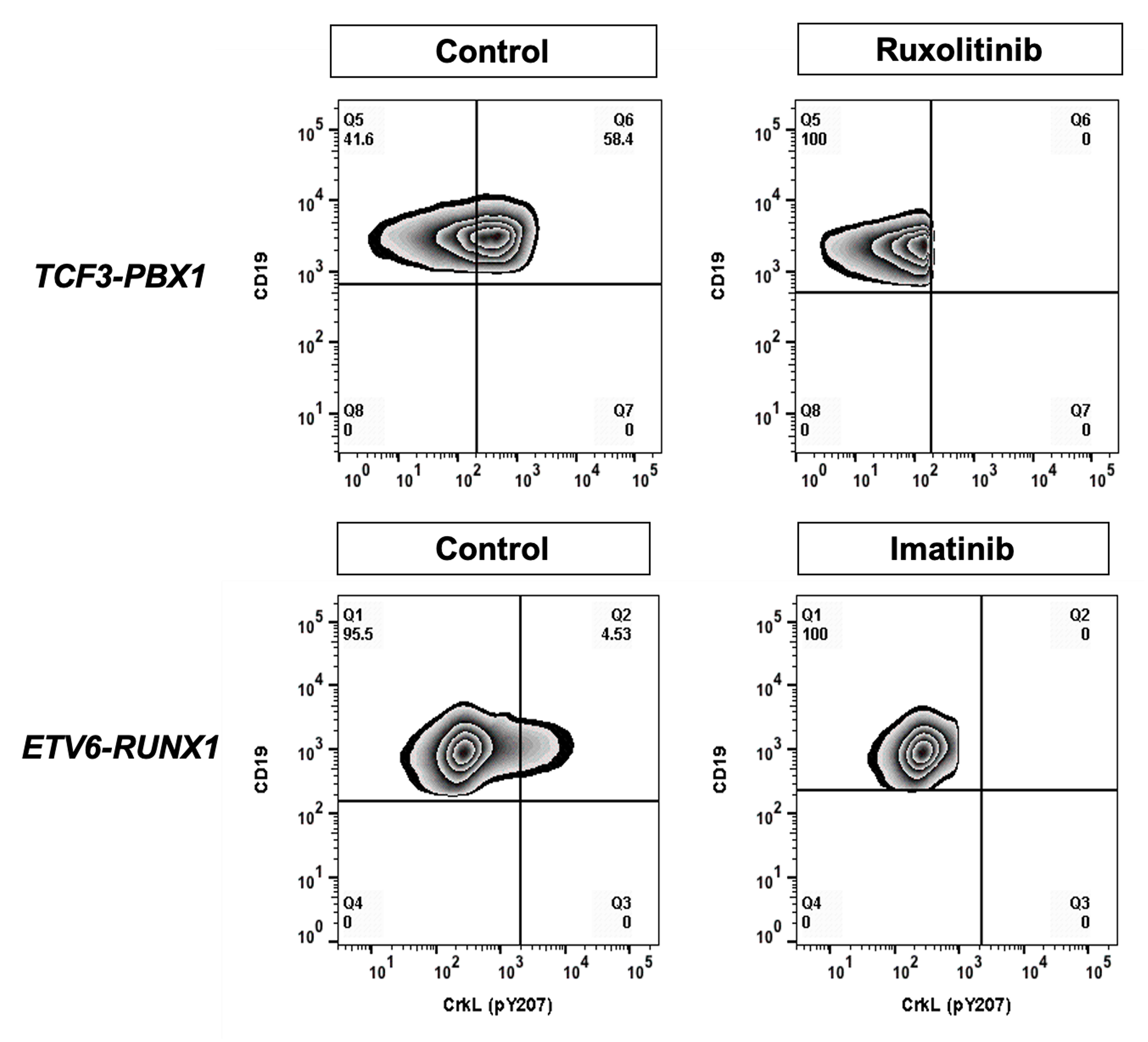


**Figure S1.** Phosphoflow analysis of the *in vitro* inhibition assay of Jak2/Stat5 and ABL pathways in blast populations from *TCF3-PBX1* and *ETV6-RUNX1* patients


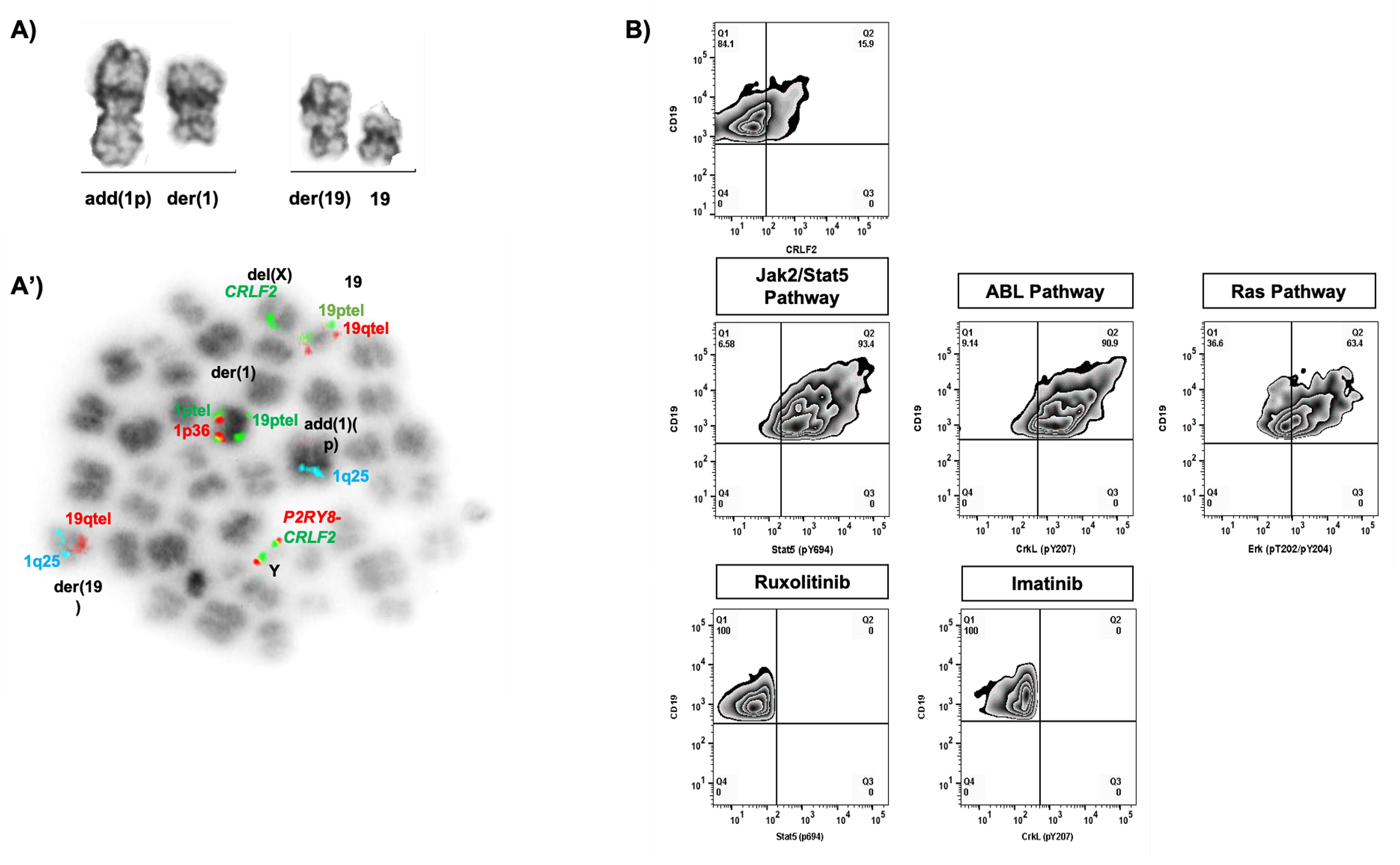


**Figure S2.** Patient with *TCF3-PBX1* at relapse. A) Partial karyotype showing chromosomes add(1)(p), der(1), der(19) and 19. A’) Metaphase with concurrence of t(1;19) and *P2RY8-CRLF2* detected by FISH. The metaphase was hybridized with 1ptel (green)/1p36 (red)/1q25 (aqua) probes, subtelomeres of chromosome 19p (green) and 19q (red), and *P2RY8-CRLF2* probe (red-green). The der(1) and der(19) present interchanged segments, in add(1)(p) 1ptel and 1p36 regions are lost, and del(X) lacks *P2RY8*. B) CRLF2 surface protein, and activation of Jak2/Stat5, ABL, and Ras pathways. Jak2/Stat5 and ABL pathways were inhibited by Ruxolitinib and Dasatinib, respectively.

**Table S1.** B-ALL patients with coexistence of *CRLF2* abnormalities and gene fusions reported in the literature and in this study.


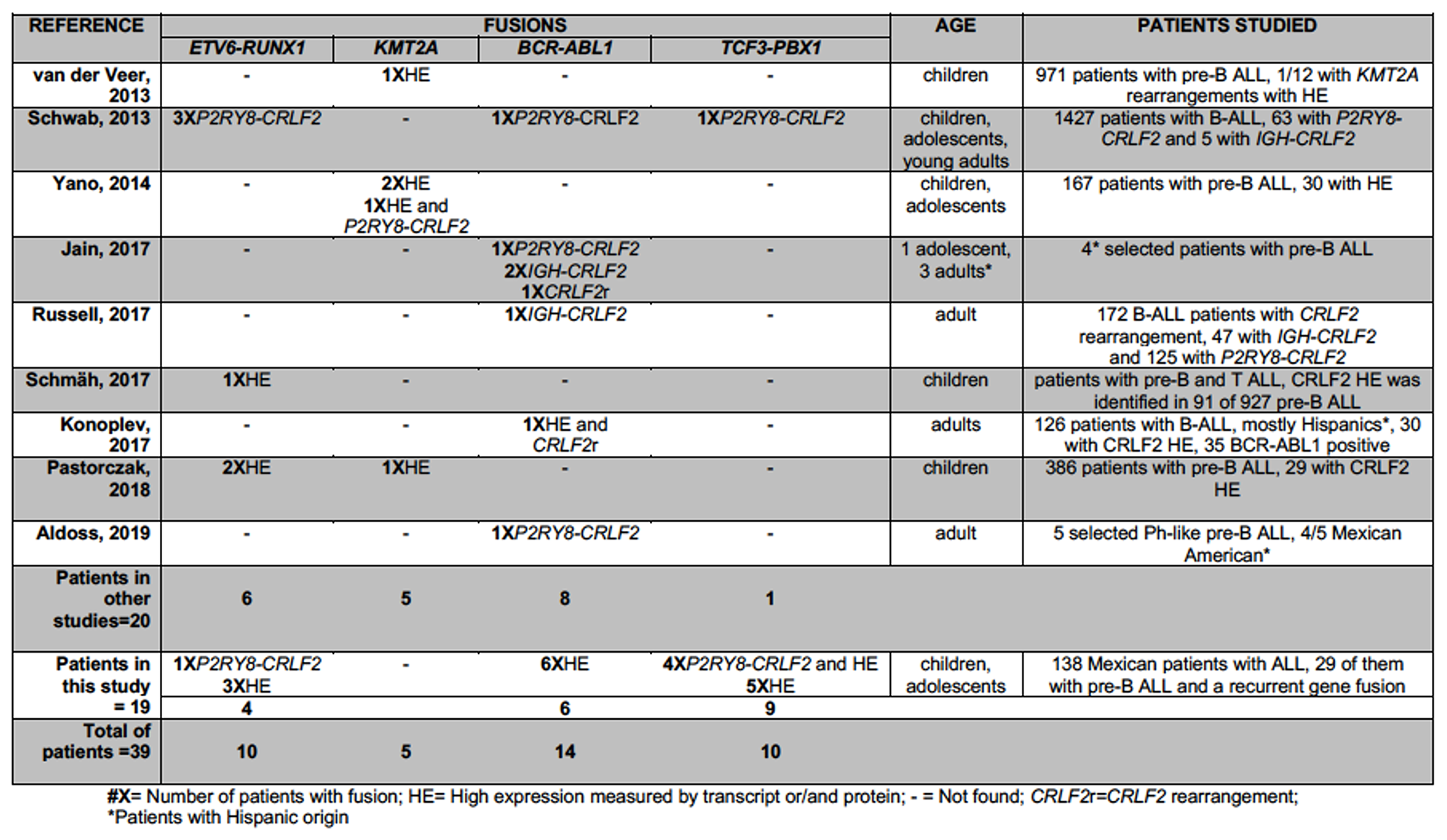

Supplement: Supplementary file 1 — Figure S1. Phosphoflow analysis of the in vitro inhibition assay of Jak2/Stat5 and ABL pathways in blast populations from TCF3‐PBX1 and ETV6‐RUNX1 patients Figure S2. Patient with TCF3‐PBX1 at relapse Table S1. B‐ALL patients with coexistence of CRLF2 abnormalities and gene fusions reported in the literature and in this study [file CJP2-7-410-s001.docx]
